# Improving nutrition through carbon reduction policies: an online randomized experiment

**DOI:** 10.1093/eurpub/ckac129.415

**Published:** 2022-10-25

**Authors:** C Law, N Berger, M Faccioli, CA Caine, IJ Bateman, RD Smith

**Affiliations:** 1 School of Agriculture Policy and Development, University of Reading, Reading, UK; 2 Population Health Innovation Lab, LSHTM, London, UK; 3 Department of Epidemiology and Public Health, Sciensano, Brussels, Belgium; 4 School of International Studies, University of Trento, Trento, Italy; 5 Department of Economics, University of Exeter Business School, Exeter, UK; 6 Exeter University Law School, Exeter University, Exeter, UK; 7 University of Exeter Medical School, Exeter, UK

## Abstract

**Background:**

There has been increasing policy interest in changing dietary patterns to reduce diet-related diseases and improve population health. Meanwhile, the food choices people make every day have a determining impact on the climate change, with food systems responsible for a third of global greenhouse gas emissions. Current policies focused on dietary health are designed, implemented and evaluated in relative isolation, and there is a critical open question concerning the extent of possible synergy with an additional focus on carbon removal.

**Methods:**

We analysed the changes in UK households’ food purchases from an online, randomized control experiment (n = 3933) designed to contrast respondents’ current food purchase behaviour with that under a range of potential tax and labelling policies targeting improvement in dietary health, alone or combined with those designed to reduce carbon emissions. We assessed changes in the healthiness of food baskets between interventions through indicators of: i) purchase of calories; ii) % of calories purchased from 23 food groups; and iii) relative changes in nutrient composition of food purchased.

**Results:**

Food labelling and fiscal measures for both health and decarbonisation have a positive impact on dietary health, by reducing the calorie content of food purchases (p < 0.001). Adding carbon reduction considerations into health policies achieves nutritional improvement by further reducing fat and increasing fibre, resulting in a reduction of up to 193 kcal/person/day (95%CI: 172-214).

**Conclusions:**

With an additional focus on planetary health, the combined (health + carbon) tax and food labelling policies could achieve a reduction in calorie content at a magnitude close to the Public Health England's estimate of average excess calories consumed by adults (195kcal).

**Key messages:**

• Policies focused on achieving both nutrition and carbon reduction goals could achieve greater improvements in food choices and produce win-win scenarios.

• There is a need for greater dialogue and policy development between public health and environmental researchers, practitioners and policy makers.

